# Molecular Characterization of Peripheral Extracellular Vesicles in Clinically Isolated Syndrome: Preliminary Suggestions from a Pilot Study

**DOI:** 10.3390/medsci5030019

**Published:** 2017-09-18

**Authors:** Nicoletta Nuzziello, Maria Blonda, Flavio Licciulli, Sabino Liuni, Antonella Amoruso, Alessio Valletti, Arianna Consiglio, Carlo Avolio, Maria Liguori

**Affiliations:** 1 National Research Council of Italy, Institute of Biomedical Technologies, Section of Bari, via Amendola no. 122/D, 70126 Bari, Italy; nicoletta.nuzziello@gmail.com (N.N.); flavio.licciulli@ba.itb.cnr.it (F.L.); sabino.liuni@ba.itb.cnr.it (S.L.); alessio.valletti@gmail.com (A.V.); ariannaconsiglio@gmail.com (A.C.); 2 Department of Basic Sciences, Neurosciences and Sense Organs, University of Bari, 70126 Bari, Italy; 3 Department of Medical and Surgical Sciences, University of Foggia, 71121 Foggia, Italy; maria.blonda@unifg.it (M.B.); antonella.amoruso@unifg.it (A.A.); carlo.avolio@unifg.it (C.A.)

**Keywords:** extracellular vesicles (EVs), Clinically Isolated Syndrome (CIS), small RNA sequencing, circulating biomarkers, bioinformatics

## Abstract

Extracellular vesicles (EVs), nanoparticles originated from different cell types, seem to be implicated in several cellular activities. In the Central Nervous System (CNS), glia and neurons secrete EVs and recent studies have demonstrated that the intercellular communication mediated by EVs has versatile functional impact in the cerebral homeostasis. This essential role may be due to their proteins and RNAs cargo that possibly modify the phenotypes of the targeted cells. Despite the increasing importance of EVs, little is known about their fluctuations in physiological as well as in pathological conditions. Furthermore, only few studies have investigated the contents of contemporary EVs subgroups (microvesicles, MVs and exosomes, EXOs) with the purpose of discriminating between their features and functional roles. In order to possibly shed light on these issues, we performed a pilot study in which MVs and EXOs extracted from serum samples of a little cohort of subjects (patients with the first clinical evidence of CNS demyelination, also known as Clinically Isolated Syndrome and Healthy Controls) were submitted to deep small-RNA sequencing. Data were analysed by an in-home bioinformatics platform. In line with previous reports, distinct classes of non-coding RNAs have been detected in both the EVs subsets, offering interesting suggestions on their origins and functions. We also verified the feasibility of this extensive molecular approach, thus supporting its valuable use for the analysis of circulating biomarkers (e.g., microRNAs) in order to investigate and monitor specific diseases.

## 1. Introduction

Extracellular vesicles (EVs), especially microvesicles (MVs, range diameter: 100–1000 nm) and exosomes (EXOs, range: 40–100 nm), are small membranous vesicles released by most cells that have been demonstrated to play a key role as mediators of several cellular functions. In the Central Nervous System (CNS), both glia and neurons secrete EVs; EVs-mediated intercellular communications seem to significantly influence the cerebral homeostasis, i.e., by impacting myelin formation, metabolic support and immune defence [1,2]. This wide range of activities may be due to the EVs cargo (RNAs, DNAs, proteins, lipids) that possibly modifies the phenotypes of the targeted cells [3]. Despite the increasingly recognized relevance of EVs, the mechanism(s) responsible for their release, as well as the characterization of the RNA contents (evRNA) in both EXOs and MVs, still need to be fully elucidated. In particular, evRNAs have been investigated especially by array-based studies [4], whereas data from high-throughput next-generation sequencing (HT-NGS) approaches are mostly derived from cultured cells or human urine [5] (Supplementary Table S1).

The implication of EVs in the pathogenesis of neurological disorders, such as Alzheimer’s, Parkinson’s diseases and stroke, has been already reported [6,7,8,9]. In CNS inflammatory demyelination like Multiple Sclerosis (MS), EVs showed both protective and damaging functions (respectively: by inducing the maturation and migration of oligodendrocyte precursor cells, and by promoting transendothelial migration of lymphocytes and monocytes that spreads the inflammation into CNS) [10]. Furthermore, the amount of MVs in cerebrospinal fluid (CSF) does change during the different clinical phases of MS, as well as compared to healthy controls (HC) or patients with other non-inflammatory disorders [11], suggesting that they may also be involved in the phenotypic expressions of the disease. So far, no published data are available on EVs profiles (in serum or CSF) of patients presenting the first clinical evidence of CNS demyelination, also known as Clinically Isolated Syndrome (CIS) that is considered the early stage of MS [12]. Indeed, the overproduction of EVs in CSF seems to be mirrored in higher numbers of EVs released by platelets, leukocytes, and monocytes in the blood of MS patients compared to HC [13]. Since peripheral blood represents an immense source of EVs (serum contains about 3 × 10^6^ EXOs/µL) [14], the analysis of its EVs contents certainly offers the advantage of easy accessibility compared to CSF.

On this ground, the main aim of this pilot study was to assess the feasibility and efficiency of EVs isolation followed by HT-sequencing of evRNAs extracted from serum samples of CIS subjects submitted to routinely clinical and instrumental follow-ups. To achieve this goal, we analysed a small cohort of CIS patients and HC subjects; the future purpose will be to apply the resulting experience to finalized scientific investigations on the role of EVs in the MS pathogenesis and its phenotypic characterization/monitoring.

## 2. Materials and Methods

### 2.1. Study Population

Five subjects, two CIS patients *naïve* for any disease-modifying therapy and three age-matched HC, were analysed. They were three females, two males (mean age 32.4 ± 12.1 years). The study was conducted in accordance with the Declaration of Helsinki: informed consent was obtained from all individual participants included in the study (approved by the Ethical Committee of the University of Foggia, Italy—prot. 120/CE/2016).

### 2.2. Extracellular Vesicles Purification

Ten mL/each of peripheral blood samples was obtained. MVs and EXOs were isolated by standardized differential centrifugations [15,16]: 10 mL of peripheral blood samples were collected in Vacutainer blood collection tubes (BDInc, Franklin Lakes, NJ, USA) and centrifuged at 3000 *g* for 7 min to separate serum fraction from blood clot. Each sample was diluted 1:1 with phosphate buffer saline (PBS) and centrifuged at 2000 × *g* for 30 min at 4 °C to eliminate cell debris and other particles. The supernatant (SN) was carefully removed, filtered through a 0.8 µm filter and centrifuged at 12,000 × *g* for 45 min at 4 °C for collecting MVs. The SN was again collected and filtered through 0.2 µm filters; EXOs were pelleted from the filtered SN by ultracentrifugation at 120,000 × *g* for 70 min at 4 °C. All the EVs were re-suspended in 200 µL PBS. 

Size distribution of MVs and EXOs was measured by the dynamic light scattering (DLS) technique with Zetasizer Nano-ZS system (Malvern Instruments, Worcestershire, UK). Ten µL of EVs were diluted in 990 µL of PBS (1:100) and then gently mixed to provide a homogenous solution. Three measurements were acquired per aliquot, with each acquisition taking 20 s. The data were acquired and analysed using Malvern Zetasizer Software (v7.11). DLS measurements allowed to discriminate between MVs and EXOs according to their different size.

### 2.3. Total RNA Isolation

Total RNA was extracted using Total Exosome RNA and Protein Isolation Kit (Invitrogen, Life Technologies, Carlsbad, CA, USA) following the manufacturer’s protocol. Ten picogramms of synthetic miRNA spike-in controls (cel-miR-39 and UniSp6) were added to the respective lysis/denaturant buffer. RNA samples were stored at −80 °C until used. RNA quantity and quality were assessed by capillary electrophoresis (Agilent 2100 Bioanalyzer, Agilent Technologies, Santa Clara, CA, USA) with total RNA 6000 Nano Chip and Small RNA chip following the manufacturer’s instructions.

### 2.4. Small RNA Deep Sequencing

Total RNA, including the small-RNA fraction, was converted into cDNA libraries using the SMARTer smRNA-Seq Kit for Illumina (Clontech Laboratories, Takara Bio Inc., Mountain View, CA, USA). Total RNA was polyadenylated in order to provide a priming sequence for an oligo(dT) primer. cDNA synthesis was primed by the 3′-smRNA-dT Primer, which incorporated an adapter sequence at the 5′-end of each first-strand cDNA molecule. PrimeScript™ Reverse Transcriptase was added at the 5′-end non-templated nucleotides, which were used as a template for the addition of a second adapter sequence to the 3′-end of each first-strand cDNA molecule. Full-length Illumina adapters were added during PCR amplification. All libraries were checked for quality (Bioanalyzer 2100 with High sensitivity DNA chip, Agilent Technologies), fluorimetrically quantified (PicoGreen Assay, Nanodrop 3300, Invitrogen, Life Technologies), then sequenced by an Illumina HiSeq2500 platform that generates 50 bp single-reads.

### 2.5. Bioinformatic and Statistical Analysis of Small RNA Data

The complete small (s)RNA-Seq analysis was performed by an in-house developed bioinformatics pipeline. Several steps compose the pipeline: raw reads filtering, reads mapping and classification, quantification [17]. The first step evaluates and filters down those reads with low quality scores using FastQC package (http://www.bioinformatics.babraham.ac.uk/projects/fastqc/) and trims adapter sequences and poly-A using cutadapt [18]. After pre-processing, read-length distribution is computed and reads >15 nucleotides were aligned against a non-redundant non-coding RNA (ncRNA) reference database (ncRNAdb http://ncRNAdb.ba.itb.cnr.it) that collects and integrates sequences and annotations of various ncRNA classes such as micro RNA (miRNA), piwi-interacting RNA (piRNA), tRNA, small nucleolar RNA (snoRNA), etc., from several public databases. In this step, two mapping tools such as Bowtie [19] and miRDeep2 (for miRNA class) [20] were applied. For completeness, unmapped reads were aligned to the Human Genome (GRCh38). Using the annotation present in ncRNAdb, the identified reads were classified in different classes of ncRNA. Finally, the quantification of the expression was computed using RSEM (RNA-Seq by Expectation Maximization). This method allocates multi-mapping reads and outputs within-sample normalized values that are corrected for sequencing biases.

## 3. Results

Considering the explorative nature of this study and the small sample size, here we reported some preliminary results and comments about individual (MV/EXO) data without statistical comparisons between the subjects’ groups.

The deep sequencing of MVs and EXOs returned raw reads counting from 12,031,509 to 57,568,091. Table 1 also shows the so-called *clean reads*, which means mapped and unmapped non-coding RNAs (ncRNAs) that resulted after removing: low-quality reads, adaptor sequences, poly-A fragments, reads smaller than 15 nucleotides, and those reads that matched mitochondrial RNAs and poorly represented ncRNAs classes (e.g., guide_RNA). After mapping the clean reads to miRBase (Release v.21) in order to annotate known miRNAs, data showed that miRNAs fraction accounted for 17.62% in CIS-1 compared to a mean of 1.75% in HC1-2 (MVs fractions), whereas in EXOs they were 2.12% in HC-3 and 0.91% in CIS-2 (Table 1).

Among the known published sequences, we found 763 (in HC-3) and 608 (in CIS-2) different mature miRNAs in EXOs samples, compared to a mean of 569 (in HC-1-2) and 940 (in CIS-1) miRNAs in MVs samples. One-hundred and eighty-two mature miRNAs were shared in all EV samples; interestingly, MVs contained six unique mature miRNAs that were absent in EXOs (hsa-miR-639, hsa-miR-4488, hsa-miR-132-5p, hsa-miR-3144-3p, hsa-miR-4519, hsa-miR-19b-1-5p) suggesting that they may be distinctive of MVs. Similarly, 34 unique mature miRNAs were exclusively present in EXO samples; five of them (hsa-miR-197-5p, hsa-miR-1262, hsa-miR-3651, hsa-miR-4492, hsa-miR-4687-3p) have been previously found to be consistently enriched in EXOs compared to cellular profiles [21,22].

**Figure 1:**
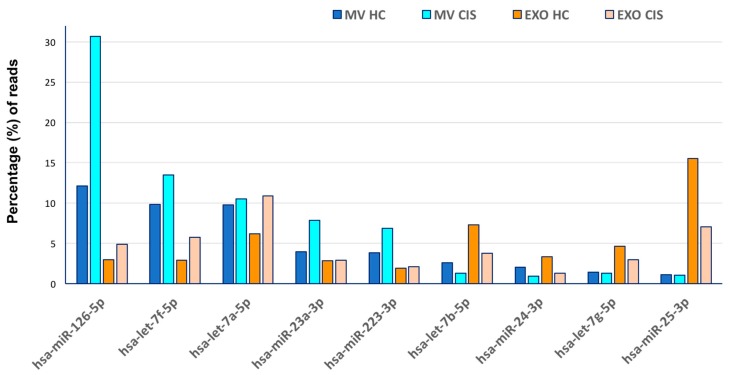
Distribution of the nine common miRNAs in the four subgroups. Data referring to MVs samples in HC-1 and HC-2 subjects are pooled together. *Y*-axe indicates the percentages of each miRNA in MVs and EXOs subgroups (CIS and HC samples).

In Table 2, the 20 most abundant miRNAs in the MV and EXO libraries have been detailed, accounting for 74.68% (MVs-HC-1-2), 86.82% (MVs-CIS-1), 77.18% EXOs-HC-3 and 71.45% (EXOs-CIS-2) of all detectable miRNAs. Nine of them were in common between the five samples (both in MVs and in EXOs aliquots); of note, the expression of hsa-miR-126-5p, hsa-let-7f-5p, hsa-let-7a-5p, hsa-miR-23a-3p and hsa-miR-223-3p was found higher in CIS samples than in HC, especially in the MVs fractions; the remaining four (hsa-let-7b-5p, hsa-miR-24-3p, hsa-let-7g-5p and hsa-miR-25-3p) seem to follow an opposite trend (Figure 1).

Table 2 also summarized the most representative ncRNAs classes. The composition of EV libraries revealed that MV and EXO samples contained piRNA, Y-RNA, miscellaneous RNA (miscRNA) and very small proportions of snoRNA, small nuclear RNAs (snRNA), tRNA, signal recognition particle RNA (srpRNA). A small percentage of rRNA seems to characterize the MV samples (2.82%) compared to the higher percentage in EXOs (9.16%), as reported by other studies [5]. However, in our view, the most interesting finding was the detection of snRNAs in both the EVs subtypes (0.09% in MVs, 0.36% in EXOs), considering that most of the published RNA species in EVs were considered of cytoplasmic origin [23]. On the contrary, the identification of long ncRNA (lncRNA) fragments (1.57% in MVs; 0.93% in EXOs) and long intergenic ncRNA (lincRNA) fragments (3.71% and 5.12%, respectively) was consistent with the cytoplasmic origin of EVs, since it is well known that RNA decay takes place in the cytoplasm [24].

Finally, small amounts of reads remained unmapped to ncRNA sequences in each of the two EVs categories; it was distinguished whether they mapped or failed to map the Human Genome sequences (GRCh38) (means and percentages are shown in Table 1). Of note, the failure to align the human genome suggests the possibility that evRNAs may transmit (or spread) genetic material of different origins, including viral, bacterial or other species, as previously reported [25].

## 4. Discussion

This study is the pilot step of a more extensive investigation that we are planning to perform in order to identify molecular and proteomic profiles of contemporary MVs and EXOs derived from serum of MS patients with different clinical phenotypes. As it was clearly reported (in the manuscript and the Tables), some of the NGS data of both EXOs and MVs were excluded from the analysis for technical reasons (mostly due to failure in the RNA extraction); we are now aware of this first crucial step, so we are quite confident of avoiding this inconvenience in the future (see take-home messages). Nevertheless, despite the individual differences that necessarily characterize the examined EVs (numbers, contents, status) and the small sample size, to our view, the overall results confirmed that sRNA-deep sequencing represents a valuable and feasible approach for the molecular characterization of EVs.

In line with previous reports [4,14,21,26], distinct classes of ncRNAs have been detected in both the EVs subsets, which contribute to improving the knowledge about EVs origins and functions (as mentioned in the previous section). On the other hand, the reported differences, e.g., in the expression levels of ncRNA categories such as miRNAs between CIS and HC subjects, might be considered distinctive of each of the two conditions, if confirmed in larger populations, thus justifying the increasing interest towards these circulating miRNAs as valuable sensitive biomarkers also in the early stage of MS.

Recently, an extensive analysis of the total circulating exosome transcriptome identified a distinct molecular profile in Relapsing-Remitting MS patients; the attention pointed towards four miRNAs (hsa-miR-122-5p, hsa-miR-532-5p, hsa-miR-196b-5p and hsa-miR-301a-3p) that were significantly decreased during clinical and MRI activity and compared to HC [26]. Although in a limited EXOs comparison (“head-to-head” CIS versus HC), we confirmed this trend, whereas in MVs samples the opposite direction was followed for the same miRNAs (*data available, not shown*). Since, in our study, we reported the same difference (MVs *versus* EXOs) for several miRNAs, we believe that the extension of this study will also help to shed light on the functional meaning of these circulating biomarkers by looking at their expression levels in contemporary MVs and EXOs.

On the other hand, the identified nine common miRNAs out of the most abundant 20 (more than 70% of the entire miRNAs in all samples) showed a peculiar opposite trend between the subgroups. Preliminary pathways analysis revealed that they were mostly involved in immunological/inflammatory processes, as well as in neurodegenerative processes. Of interest, hsa-miR-24-3p has already been reported up-regulated in Primary Progressive MS compared to Secondary Progressive MS and HC [27], whereas miR-25-3p, let-7f-5p, miR-23a-3p, miR-223-3p and the same miR-24-3p were found functionally related to EVs transfer or senescence [28,29,30,31,32,33], and miR-19b-3p was identified in EXOs of AD subjects [33].

In conclusion, although with caution due to the small sample size, we believe that the data produced by this pilot observation do represent a start point for future in-depth analysis investigating the implications of EVs in MS pathogenesis. At the same time, it is plausible that circulating EVs may represent a specific target for novel treatments, leading to the identification of additional (and possibly more effective) therapeutic approaches.

## 5. Take-Home Messages

1. The analysis of EVs contents (especially sRNAs) may help to provide more information on their functional role(s) in several diseases; EVs contents may also be used for the isolation of circulating biomarkers (e.g., miRNAs).
2. Few studies have investigated both the EV classes (Microvesicles and Exosomes) extracted simultaneously from the peripheral blood samples in the same patient, but this strategy should be suggested in order to fully understand the entire molecular picture of a given status.
3. smallRNA deep sequencing is a valuable approach for studying the different classes of small non-coding RNA, although a solid bioinformatics support is recommended for the analysis and the interpretation of the obtained results.
4. In order to obtain the right amount of evRNAs for the subsequent sRNA-seq analysis, we suggest/recommend to collect a higher quantity of blood samples (at least 20 mL/each) or to pool together samples belonging to different subjects with the same phenotype [34].

## Figures and Tables

**Table 1:** Summary of the most representative small-RNA libraries in the examined extracellular vesicles (EVs) samples (no. 3 for microvesicles (MVs), no. 2 for exosomes (EXOs)).

	Microvesicles					Exosomes				
	HC-1		HC-2		CIS-1		HC-3		CIS-2	
	Read Count	%	Read Count	%	Read Count	%	Read Count	%	Read Count	%
Raw reads	12,031,509		26,023,784		23,176,535		57,568,091		17,246,086	
Clean reads (mapped/unmapped)	3,851,795		18,679,042		7,845,182		39,163,534		10,030,669	
**Mapped ncRNA**										
miRNA	73,459	1.91	143,322	0.77	1,382,492	17.62	829,102	2.12	91,160	0.91
miRNA_primary_transcript	13,089	0.34	12,758	0.07	66,145	0.84	5,419,917	13.84	520,722	5.19
piRNA	70,927	1.84	497,413	2.66	653,901	8.34	1,587,896	4.05	172,016	1.71
misc_RNA	52,601	1.37	273,814	1.47	119,722	1.53	178,440	0.46	77,591	0.77
rRNA	34,888	0.91	786,417	4.21	36,569	0.47	3,971,869	10.14	534,541	5.33
snoRNA	754	0.02	1330	0.01	3442	0.04	72,452	0.18	15,955	0.16
snRNA	2091	0.05	14,445	0.08	11,740	0.15	95,877	0.24	83,351	0.83
SRP_RNA	2810	0.07	22,088	0.12	7666	0.10	10,246	0.03	829	0.01
tRNA	12,925	0.34	465,531	2.49	42,955	0.55	276,975	0.71	23,337	0.23
vault_RNA	106	0.001	2331	0.01	793	0.001	14,463	0.04	2120	0.02
Y_RNA	27,020	0.70	276,914	1.48	454,862	5.80	390,174	1.00	31,963	0.32
antisense	28,863	0.75	90,018	0.48	44,561	0.57	347,191	0.89	95,003	0.95
lincRNA	573,422	14.89	215,484	1.15	338,285	4.31	1,227,887	3.14	1,289,945	12.86
lncRNA	158,954	4.13	214,994	1.15	102,465	1.31	279,388	0.71	177,791	1.77
processed_transcript	134,450	3.49	268,735	1.44	128,558	1.64	264,513	0.68	121,479	1.21
retained_intron	102,548	2.66	180,506	0.97	126,455	1.61	530,026	1.35	228,149	2.27
**Unmapped ncRNA**										
Human genome (GRCh38)	856,079	22.23	1,643,508	8.80	1,975,727	25.18	16,540,083	42.23	5,153,999	51.38
unmapped Human genome	1,706,809	44.31	13,569,434	72.65	2,348,844	29.94	7,127,035	18.20	1,410,718	14.06

HC: healthy controls; CIS: Clinically Isolated Syndrome; miRNA: microRNA; piRNA: piwi-interacting RNA; miscRNA: miscellaneous RNA; snoRNA: small nucleolar RNA; snRNA: small nuclear RNAs; srpRNA: signal recognition particle RNA; lincRNA: long intergenic ncRNA; lncRNA: long noncoding RNA.

**Table 2:** The 20 most abundant miRNAs in MVs and EXOs samples, accounting for 74.68% (in MVs-HC), 86.82% (MVs-CIS), 77.18% EXOs-HC) and 71.45% (EXOs-CIS) of all detectable miRNAs. In bold, nine miRNAs that are in common in all EV samples.

MVs						EXOs					
HCs-1-2			CIS-1			HC-3			CIS-2		
Transcript ID	Read Count *	%	Transcript ID	Read Count	%	Transcript ID	Read Count	%	Transcript ID	Read Count	%
hsa-miR-126-5p	33,125.28	12.11	hsa-miR-126-5p	775,815	30.67	hsa-miR-25-3p	129,373	15.46	hsa-let-7a-5p	24,658	10.88
hsa-let-7f-5p	26,785.99	9.80	hsa-let-7f-5p	341,213	13.49	hsa-let-7b-5p	60,980	7.29	hsa-miR-25-3p	15,906	7.02
hsa-let-7a-5p	26,706.14	9.77	hsa-let-7a-5p	266,308	10.53	hsa-miR-451a	60,215	7.20	hsa-miR-3908	15,510	6.84
hsa-miR-142-3p	16,230.72	5.94	hsa-miR-23a-3p	199,088	7.87	hsa-let-7a-5p	51,475	6.15	hsa-let-7f-5p	13,026	5.75
hsa-miR-1246	15,182.89	5.55	hsa-miR-223-3p	172,699	6.83	hsa-let-7g-5p	38,560	4.61	hsa-miR-126-5p	11,069	4.88
hsa-miR-126-3p	11,081.05	4.05	hsa-miR-150-5p	113,228	4.48	hsa-miR-19b-3p	28,400	3.39	hsa-miR-5096	10,094	4.45
hsa-miR-23a-3p	10,809.96	3.95	hsa-miR-151a-3p	34,022	1.35	hsa-miR-24-3p	28,108	3.36	hsa-let-7b-5p	8506	3.75
hsa-miR-223-3p	10,415.68	3.81	hsa-let-7b-5p	33,279	1.32	hsa-miR-1246	26,006	3.11	hsa-miR-1273c	7323	3.23
hsa-miR-150-5p	10,384.42	3.80	hsa-let-7g-5p	32,259	1.28	hsa-miR-126-5p	24,775	2.96	hsa-miR-8086	6988	3.08
hsa-miR-1260b	8540.57	3.12	hsa-miR-146a-5p	26,769	1.06	hsa-miR-122-5p	24,611	2.94	hsa-let-7g-5p	6770	2.99
hsa-let-7b-5p	7040.47	2.57	hsa-miR-25-3p	26,213	1.04	hsa-let-7f-5p	24,091	2.88	hsa-miR-23a-3p	6519	2.88
hsa-miR-191-5p	5648.99	2.07	hsa-miR-24-3p	23,816	0.94	hsa-miR-23a-3p	23,673	2.83	hsa-miR-1290	6292	2.78
hsa-miR-24-3p	5522.51	2.02	hsa-miR-1260b	23,499	0.93	hsa-miR-486-5p	23,461	2.80	hsa-miR-7704	5136	2.27
hsa-let-7g-5p	3832.22	1.40	hsa-miR-126-3p	22,682	0.90	hsa-miR-92a-3p	21,203	2.53	hsa-miR-223-3p	4713	2.08
hsa-miR-25-3p	3070.25	1.12	hsa-miR-21-5p	21,229	0.84	hsa-miR-223-3p	15,895	1.90	hsa-miR-1303	3914	1.73
hsa-miR-146a-5p	2287.37	0.84	hsa-miR-191-5p	20,705	0.82	hsa-miR-486-3p	13,892	1.66	hsa-miR-4279	3488	1.54
hsa-miR-92a-3p	2115.47	0.77	hsa-miR-92a-3p	18,397	0.73	hsa-miR-16-5p	13,286	1.59	hsa-miR-6087	3312	1.46
hsa-miR-151a-3p	1937.60	0.71	hsa-let-7d-3p	17,960	0.71	hsa-miR-191-5p	12,984	1.55	hsa-miR-19b-3p	3103	1.37
hsa-miR-342-3p	1760.54	0.64	hsa-miR-23b-3p	14,808	0.59	hsa-miR-93-5p	12,851	1.54	hsa-miR-24-3p	2979	1.31
hsa-miR-486-3p	1706.72	0.62	hsa-miR-374b-5p	12,218	0.48	hsa-miR-19a-3p	11,956	1.43	hsa-miR-6087	2622	1.16

* Mean of the read counts between the two HC samples; the percentage has been calculated on the resulted data

## Supplementary Materials

The following are available online at www.mdpi.com/2076-3271/5/3/19/s1, Table S1: Published studies on EVs sequencing performed by HT-NGS.
