# Supplementary material for: Molecular Characterization of Peripheral Extracellular Vesicles in Clinically Isolated Syndrome: Preliminary Suggestions from a Pilot Study
Source: Med Sci (Basel). 2017 Sep 18;5(3):19. doi: 10.3390/medsci5030019 (PMC5635803; doi:10.3390/medsci5030019)
Supplement: Supplementary file 1 [file medsci-05-00019-s001.docx]

| **Reference** | **Sample** | **Next Generation Sequencing system** |
| --- | --- | --- |
| da Silveira, J.C.; Andrade, G.M.; del Collado, M. et al. Supplementation with small-extracellular vesicles from ovarian follicular fluid during in vitro production modulates bovine embryo development. PLoS One **2017**, 12, e0179451 | EXOs from bovine ovarian follicular fluid | HiScanSQ platform (Illumina) |
| Conley, A.; Minciacchi, V.R.; Lee, D.H. et al. High-throughput sequencing of two populations of extracellular vesicles provides an mRNA signature that can be detected in the circulation of breast cancer patients. RNA Biology **2017**, 14, 305-316 | MVs and EXOs from human plasma and breast cancer cells | GAIIx platform (Illumina) |
| Lässer, C.; Shelke, G.V.; Yeri, A. et al. Two distinct extracellular RNA signatures released by a single cell type identified by microarray and next-generation sequencing. RNA Biology **2017**, 14, 58-72 | EXOs from human mast (HMC-1) and erythroleukemic  (TF-1) cell lines | HiSeq 2500 platform (Illumina) |
| Tsang, E.K.; Abel, N.S.; Li, X. et al. Small RNA Sequencing in Cells and Exosomes identifies eQTLs and 14q32 as a region of active export. G3 **2017**, 7, 31-39. | EXOs from lymphoblastoid (LCL) cell line | HiSeq 2000 platform (Illumina) |
| Choi, J.W.; Kim, S.C.; Hong, S.H. et al. Secretable Small RNAs via outer membrane vesicles in periodontal pathogens. J Dent Res **2017**, 10.1177/0022034516685071 | EXOs from peridontal pathogens | HiSeq 2000 platform (Illumina) |
| van Eijndhoven, M.A.J.; Zijlstra, J.M.; Groenewegen, N.J. et al. Plasma vesicle miRNAs for therapy response monitoring in Hodgkin lymphoma patients. JCI Insight. **2016**, 1, e89631 | EVs from human plasma | HiSeq 2500 platform (Illumina) |
| Ogawa, Y.; Tsujimoto, M.; Yanoshita, R. Next-Generation Sequencing of protein-coding and long non-protein coding RNAs in two types of exosomes derived from human whole saliva. Biol. Pharm. Bull. **2016**, 39, 1496-1507 | EXOs from human saliva | GAIIx platform (Illumina) |
| Yuan, T.; Huang, X.; Woodcock, M. et al. Plasma extracellular RNA profiles in healthy and cancer patients*.* Scientific Reports. **2016**, 6, 19413 | EXOs from human plasma | HiSeq 2000 platform (Illumina) |
| Fiskaa, T.; Knutsen, E.; Nikolaisen, M.A. et al. Distinct small RNA signatures in Extracellular Vesicles derived from Breast Cancer Cell Lines. PLoS One **2016,** 11, e0161824 | EXOs from human breast cancer cell lines | SOLiD platform (Applied Biosystems) |
| San Lucas, F.A.; Allenson, K.; Bernard, V. et al. Minimally invasive genomic and transcriptomic profiling of visceral cancers by next-generation sequencing of circulating exosomes. Ann Oncol. **2016**, 27, 635-41 | EXOs from human plasma and pleural effusion | HiSeq 2500 platform (Illumina) |
| Koppers-Lalic, D.; Hackenberg, M.; de Menezes, R. et al. Non-invasive prostate cancer detection by measuring miRNA variants (isomiRs) in urine extracellular vesicles. Oncotarget. **2016**, 7, 22566-22578. | EXOs from human urine | HiSeq 2000 platform (Illumina) |
| Ben-Dov, I.Z.; Whalen, V.M.; Goilav, B. et al. Cell and Microvesicle Urine microRNA Deep Sequencing Profiles from Healthy Individuals: Observations with Potential Impact on Biomarker Studies. PLoS One. **2016**, 11, e0147249 | EVs from human urine | GAIIx platform (Illumina) |
| Au Yeung, C.L.; Co, N.N.; Tsuruga, T. et al. Exosomal transfer of stroma-derived miR21 confers paclitaxel resistance in ovarian cancer cells through targeting APAF1. Nat Commun. **2016**, 7, 11150 | EXOs from human ovarian cancer cell line | Ion Torrent platform (Applied Biosystems) |
| Sánchez, C.A.; Andahur, E.I.; Valenzuela, R. et al. Exosomes from bulk and stem cells from human prostate cancer have a differential microRNA content that contributes cooperatively over local and pre-metastatic niche. Oncotarget. **2016**, 7, 3993-4008. | EXOs from bulk and stem cells | HiSeq 2000 platform (Illumina) |
| De Luca, L.; Trino, S.; Laurenzana, I. et al. MiRNAs and piRNAs from bone marrow mesenchymal stem cell extracellular vesicles induce cell survival and inhibit cell differentiation of cord blood hematopoietic stem cells: a new insight in transplantation. Oncotarget. **2016**, 7, 6676-6692 | EXOs from bone marrow mesenchymal stem cell line | HiSeq 1000 platform (Illumina) |
| Hannafon, B.N.; Trigoso, Y.D.; Calloway, C.L. et al. Plasma exosome microRNAs are indicative of breast cancer. Breast Cancer Res. **2016**, 18, 90 | EXOs from human breast cancer cell lines | MiSeq platform (Illumina) |
| Stevanato, L.; Thanabalasundaram, L.; Vysokov, N. et al. Investigation of content, stoichiometry and transfer of miRNA from human neural stem cell line derived Exosomes. PLoS One. **2016**, 11, e0146353 | EXOs from human neural stem cell line | HiSeq 2000 platform (Illumina) |
| Lugli, G.; Cohen, A.M.; Bennett, D.A. et al, Smalheiser NR. Plasma exosomal miRNAs in persons with and without Alzheimer Disease: altered expression and prospects for biomarkers. PLoS One. **2015**, 10, e0139233 | EXOs from human plasma | HiSeq 2500 platform (Illumina) |
| Lunavat, T.R.; Cheng, L.; Kim, D.K. et al. Small RNA deep sequencing discriminates subsets of extracellular vesicles released by melanoma cells Evidence of unique microRNA cargos. RNA Biology **2015**, 12, 810-23 | MVs and EXOs from malignant melanoma (A375, MML-1 and SK-MEL-28) cell lines | Ion Torrent platform (Applied Biosystems) |
| Alexander, M.; Hu, R.; Runtsch, M.C. et al. Exosome-delivered microRNAs modulate the inflammatory response to endotoxin. Nat Commun. **2015**, 6, 7321 | EXOs from mouse primary bone marrow-derived  DCs (BMDC) | HiSeq 2000 platform (Illumina) |
| Collino, F.; Bruno, S.; Incarnato, D. et al. AKI Recovery induced by mesenchymal stromal cell-derived extracellular vesicles carrying microRNAs. J Am Soc Nephrol. **2015**, 26, 2349-2360. | EXOs from mesenchymal stromal cell line | HiScanSQ platform (Illumina) |
| Huang, X.; Yuan, T.; Liang, M. et al. Exosomal miR-1290 and miR-375 as prognostic markers in castration-resistant prostate cancer. Eur Urol. **2015**, 67, 33-41 | EXOs from human plasma | HiSeq 2000 platform (Illumina) |
| van Balkom, B.W.; Eisele, A.S.; Pegtel, D.M. et al. Quantitative and qualitative analysis of small RNAs in human endothelial cells and exosomes provides insights into localized RNA processing, degradation and sorting. J Extracell Vesicles. **2015**, 4, 26760 | EXOs from human endothelial (HMEC-1) cell line | HiSeq 2000 platform (Illumina) |
| Dismuke, W.M.; Challa, P.; Navarro, I. et al. Human aqueous humor exosomes. Exp Eye Res. **2015**, 132, 73-7 | EXOs from human aqueous humor | MiSeq platform (Illumina) |
| Baglio, S.R.; Rooijers, K.; Koppers-Lalic, D. et al. Human bone marrow- and adipose-mesenchymal stem cells secrete exosomes enriched in distinctive miRNA and tRNA species. Stem Cell Res Ther. **2015**, 6, 127 | EXOs from human bone marrow- and adipose-mesenchymal stem cells | HiSeq 2000 platform (Illumina) |
| Tosar, J.P.; Gámbaro, F.; Sanguinetti, J. et al. Assessment of small RNA sorting into different extracellular fractions revealed by high-throughput sequencing of breast cell lines. Nucleic Acids Res. **2015**, 43, 5601-5616. | MVs and EXOs from breast (MCF-7, MCF-10A, NCI-H1299) cell lines | GAIIx platform (Illumina) |
| Liao, J.; Liu, R.; Yin, L. et al. Expression Profiling of Exosomal miRNAs Derived from Human Esophageal Cancer Cells by Solexa High-Throughput Sequencing. Int. J. Mol. Sci. **2014**, 15, 15530-51 | EXOs from human esophageal cancer (EC9706) cell line | Solexa platform (Illumina) |
| Koppers-Lalic, D.; Hackenberg, M.; Bijnsdorp, I.V. et al. Nontemplated nucleotide additions distinguish the small RNA composition in cells from exosomes. Cell Rep. **2014**, 8, 1649-58 | EXOs form Epstein Barr Virus positive lymphoblastoid, EBV negative diffuse large B cell lymphoma, Burkitt’s Lymphoma cell lines | HiSeq 2000 platform (Illumina) |
| Bang, C.; Batkai, S.; Dangwal, S. et al. Cardiac fibroblast–derived microRNA passenger strand-enriched exosomes mediate cardiomyocyte hypertrophy. J Clin Invest. **2014**, 124, 2136-2146 | EXOs from rat cardiac fibroblasts | HiSeq 2000 platform (Illumina) |
| McDonald, M.K.; Tian, Y.; Qureshi, R.A. et al. Functional significance of macrophage-derived exosomes in inflammation and pain. Pain. **2014**, 155, 1527-1539 | EXOs from RAW 264.7 cells and human serum | SOLiD platform (Applied Biosystems) |
| Ji, H.; Chen, M.; Greening, D.W. et al. Deep Sequencing of RNA from Three Different Extracellular Vesicle (EV) Subtypes Released from the Human LIM1863 Colon Cancer Cell Line uncovers distinct miRNA-enrichment signatures. PLOS One **2014**, 9, e110314 | MVs and EXOs from human colon cancer (LIM1863) cell line | HiSeq 2000 platform (Illumina) |
| Miranda, K.C.; Bond, D.T.; Levin, J.Z. et al. Massively parallel sequencing of human urinary Exosome/Microvesicle RNA reveals a predominance of non-coding RNA. PLOS One **2014**, 9, e96094 | MVs and EXOs from human urine | GAII platform (Illumina) |
| Burns, G.; Brooks, K.; Wildung, M. et al. Extracellular Vesicles in luminal fluid of the ovine uterus*.* PLOS one **2014**, 9, e90913 | EXOs from uterine luminal fluid | Ion Torrent platform (Applied Biosystems) |
| Tietje, A.; Maron, K.N.; Wei, Y. et al. *C*erebrospinal Fluid Extracellular Vesicles undergo age dependent declines and contain known and novel non-coding RNAs. PLOS One **2014**, 9, e113116. | EXOs from CSF | HiSeq 2000 platform (Illumina) |
| Cheng, L.; Sharples, R.A.; Scicluna, B.J. et al. Exosomes provide a protective and enriched source of miRNA for biomarker profiling compared to intracellular and cell-free blood. J Extracell Vesicles. **2014**, 3, 23743 | EXOs from human blood | Ion Torrent PGM platform (Applied Biosystems) |
| Li, M.; Zeringer, E.; Barta, T. et al. Analysis of the RNA content of the exosomes derived from blood serum and urine and its potential as biomarkers. Philos Trans R Soc Lond B Biol Sci. **2014**, 369, 20130502 | EXOs from human urine and serum | Ion Torrent PGM platform (Applied Biosystems) |
| Huang, X.; Yuan, T.; Tschannen, M. et al. Characterization of human plasma-derived exosomal RNAs by deep sequencing. MC Genomics **2013**, 14, 319 | EXOs from human plasma | HiSeq 2000 platform (Illumina) |
| Jenjaroenpun, P.; Kremenska, Y.; Nair, V.M. et al. Characterization of RNA in exosomes secreted by human breast cancer cell lines using next-generation sequencing. PeerJ. **2013**, 1, e201 | EXOs from human breast cancer cell lines | Ion Torrent PGM platform (Applied Biosystems) |
| Bellingham, S.A.; Coleman, B.M.; Hill, A.F. Small RNA deep sequencing reveals a distinct miRNA signature released in exosomes from prion-infected neuronal cells. Nucleic Acids Research. **2012**, 40, 10937-10949 | EXOs from mouse hypothalamic neuronal (GT1-7)  cell line | SOLiD platform (Applied Biosystems) |
